# Neoadjuvant Chemotherapy with Gemcitabine Plus Nab-Paclitaxel Regimen for Borderline Resectable Pancreatic Cancer with Arterial Involvement: A Prospective Multicenter Single-Arm Phase II Study Protocol

**DOI:** 10.29337/ijsp.142

**Published:** 2021-04-26

**Authors:** Yoshihiro Miyasaka, Takao Ohtsuka, Susumu Eguchi, Masafumi Inomata, Kazuyoshi Nishihara, Hiroyuki Shinchi, Koji Okuda, Hideo Baba, Hiroaki Nagano, Toshiharu Ueki, Hirokazu Noshiro, Masafumi Nakamura, Kyushu study group of treatment for pancreatobiliary cancer

**Affiliations:** 1 Department of Surgery and Oncology, Graduate School of Medical Sciences, Kyushu University, Japan; 2 Department of Surgery, Fukuoka University Chikushi Hospital, Japan; 3 Digestive Surgery, Breast and Thyroid Surgery, Graduate School of Medical Sciences, Kagoshima University, Japan; 4 Department of Surgery, Nagasaki University Graduate School of Biomedical Sciences, Japan; 5 Department of Gastroenterological and Pediatric Surgery, Oita University Faculty of Medicine, Japan; 6 Department of Surgery, Kitakyushu Municipal Medical Center, Japan; 7 Department of Surgery, Kurume University School of Medicine, Japan; 8 Department of Gastroenterological Surgery, Graduate School of Medical Sciences, Kumamoto University, Japan; 9 Department of Gastroenterological, Breast and Endocrine Surgery, Yamaguchi University Graduate School of Medicine, Japan; 10 Department of Gastroenterology, Fukuoka University Chikushi Hospital, Japan; 11 Department of Surgery, Saga University Faculty of Medicine, Japan; 12 Japan

**Keywords:** borderline resectable pancreatic cancer, gemcitabine, nab-paclitaxel, neoadjuvant

## Abstract

**Introduction:**

Although neoadjuvant treatment is recommended for patients with borderline resectable pancreatic cancer (BRPC), no standard neoadjuvant regimen has been established for BRPC with arterial involvement (BRPC-A), which is associated with a higher risk of margin-positive resection and poorer prognosis than BRPC with only venous involvement. Gemcitabine plus nab-paclitaxel (GnP) has been reported to significantly reduce tumor size in metastatic pancreatic cancer, and some retrospective studies suggested that neoadjuvant GnP for BRPC improved resectability and survival.

**Methods and analysis:**

A prospective multicenter single-arm phase II study is conducted to evaluate the safety and efficacy of GnP as neoadjuvant chemotherapy for BRPC-A. The primary endpoint is the R0 resection rate. The secondary endpoints are the neoadjuvant chemotherapy response rate, resection rate, pathological response rate, incidence rate of adverse events, and quality of life.

**Ethics and dissemination:**

This study protocol was approved by the institutional review board of Kyushu University (no. 181). The results will be published in a peer-reviewed journal and will be presented at medical meetings.

**Highlights:**

- Strategy for borderline resectable pancreatic cancer involving arteries (BRPC-A).
- There is no standard regimen for neoadjuvant chemotherapy for BRPC-A.
- Gemcitabine plus nab-paclitaxel (GnP) shows significant tumor shrinkage.
- Neoadjuvant GnP for BRPC-A increases resectability and margin-negative resection.

## 1. Background

Borderline resectable pancreatic cancer (BRPC) is a recently proposed category that carries a high risk of margin-positive resection when it is initially treated via surgical resection, and it is defined by the involvement of major vessels, such as the celiac artery, superior mesenteric artery, hepatic artery, or portal vein (PV) [1]. Initial surgery does not efficiently improve the survival of patients with BRPC, and several retrospective studies suggested that neoadjuvant treatment for BRPC increases the R0 resection rate and improves survival [2]. The National Comprehensive Cancer Network guidelines recommend neoadjuvant treatment for BRPC [3]. However, there is no standard neoadjuvant regimen for this malignancy.

BRPC with arterial involvement (BRPC-A) is associated with a higher rate of margin-positive resection and poorer prognosis than BRPC involving the PV or superior mesenteric vein (SMV) (BRPC-PV) [2]. An analysis of patients with BRPC who received preoperative gemcitabine-based chemoradiotherapy revealed a significantly lower resection rate in patients with BRPC-A than in those with BRPC-PV [4]. Pancreatic resection combined with resection and reconstruction of the PV/SMV can be performed safely, and it is a common surgical procedure for patients with BRPC-PV [5,6]. Contrarily, pancreatic resection combined with the resection of major arteries is associated with high risks of morbidity and mortality [7]. Therefore, pancreatic resection plus arterial resection is not generally accepted as a standard surgical procedure for BRPC-A [5]. To avoid arterial resection, a treatment regimen with a significant tumor-shrinking effect is ideal for neoadjuvant treatment.

Gemcitabine plus nab-paclitaxel (GnP) was originally reported to improve the survival of patients with metastatic pancreatic cancer [8]. A substantial tumor-shrinking effect was also described for GnP [9]. A retrospective study of patients with BRPC revealed that neoadjuvant GnP was linked to a significantly higher R0 resection rate and longer survival than upfront surgery, although the proportion of patients with BRPC and arterial involvement was significantly higher in the neoadjuvant GnP group [10]. In this study, 92% of patients with BRPC-A in the neoadjuvant GnP group did not require arterial resection, and margin-free resection was achieved in all those patients. However, the efficacy of neoadjuvant GnP for BRPC-A has not been evaluated in the prospective setting. Therefore, we are conducting a prospective multicenter phase II study to evaluate the efficacy and safety of neoadjuvant GnP for BRPC-A.

## 2. Methods and Analysis

### 2.1. Objective

The primary objective of this study is to evaluate the safety and efficacy of the GnP regimen as neoadjuvant chemotherapy for BRPC-A.

### 2.2. Study design

This study is a prospective, single-arm, multicenter phase II trial with 14 participating institutions, which was registered with the UMIN Clinical Trials Registry as UMIN000027775. This study protocol was approved by the institutional review board of Kyushu University (no. 181).

### 2.3. Endpoints

The primary endpoint is the R0 resection rate. The secondary endpoints are the neoadjuvant chemotherapy response rate, resection rate, pathological response rate, incidence rate of adverse events, and quality of life endpoints.

### 2.4. Patient eligibility

#### 2.4.1. Inclusion criteria

1. Histological or cytological diagnosis of pancreatic adenocarcinoma or adenosquamous carcinoma
2. Presence of BRPC-A as defined according to the General Rules for the Study of Pancreatic Cancer 7^th^ Edition from the Japan Pancreatic Society (Tumor contact or invasion of superior mesenteric artery and/or celiac artery of less than 180 degrees without showing stenosis or deformity. Tumor contact or invasion of common hepatic artery without showing tumor contact or invasion of proper hepatic artery and/or celiac artery.) [11]
3. Presence of a measurable lesion
4. No distant metastasis
5. No prior treatment for pancreatic cancer
6. Age of at least 20 years but less than 75 years
7. Eastern Cooperative Oncology Group (ECOG) performance status of 0 or 1
8. No history of chemotherapy or radiotherapy
9. Spared organ function satisfying the following laboratory data (within 14 days before registration): white blood cell count ≤ 12,000/mm^3^; neutrophil count ≥ 1500/mm^3^; hemoglobin ≥ 9.0 g/dL; platelet count ≥ 100,000/mm^3^; aspartate aminotransferase (AST) ≤ 100 U/L; alanine aminotransferase (ALT) ≤ 100 U/L; total bilirubin ≤ 1.8 mg/dL; and creatinine ≤ 1.5 mg/dL.
10. ≤Grade 1 peripheral sensory/motor neuropathy
11. Obtained written informed consent to participate in this study

#### 2.4.2. Exclusion criteria

1. Synchronous or metachronous (within 5 years) multiple cancer excluding carcinoma in situ
2. Pregnancy or lactation in women, childbearing potential in women, or expectation of a partner’s pregnancy for men
3. Active infection
4. Mental illness that makes study participation difficult
5. Interstitial pneumonia/pulmonary fibrosis
6. Severe cardiac/liver disease or uncontrollable diabetes mellitus
7. Positivity for HBs antigen
8. Difficulty to perform enhanced computed tomography (CT) because of renal dysfunction or allergy to the contrast medium
9. Severe drug hypersensitivity
10. Inappropriateness for enrollment based on the judgment of the researchers or primary care physicians

### 2.5. Registration

After confirmation of eligibility, registration forms will be sent to the Clinical Research Support Center Kyushu (CReS Kyushu).

### 2.6. Treatment

#### 2.6.1. Neoadjuvant chemotherapy

Protocol treatment will be started within 14 days after registration. Enrolled patients will receive an intravenous infusion of 1,000 mg/m^2^ gemcitabine and 125 mg/m^2^ nab-paclitaxel on days 1, 8, and 15 of each 28-day cycle. The neoadjuvant treatment will be repeated for 2–4 cycles. The number of cycles will be decided by the physicians or surgeons of participating institutions based on laboratory data, imaging findings, and the patient’s condition. On day 1 of the first cycle, chemotherapy will be administered if patients fulfill the eligibility criteria. On days 8 and 15 of each cycle, chemotherapy will be continued if patients fulfill the following criteria: neutrophil count > 1,200/mm^3^; platelet count ≥ 100,000/mm^3^; AST ≤ 150 U/L; ALT ≤ 150 U/L; total bilirubin ≤ 3.0 mg/dL; ≤Grade 1 diarrhea, anorexia, nausea, vomiting, or infection; ≤Grade 2 mucositis, rash, or peripheral sensory/motor neuropathy; and no symptom suggesting macular edema. On day 1 of cycles 2–4, chemotherapy will be continued if patients fulfill the following criteria: neutrophil count > 1,000/mm^3^; platelet count ≥ 75,000/mm^3^; AST ≤ 150 U/L; ALT ≤ 150 U/L; total bilirubin ≤ 3.0 mg/dL; ≤Grade 1 diarrhea, anorexia, nausea, vomiting, or infection; ≤Grade 2 mucositis, rash, or peripheral sensory/motor neuropathy; and no symptom suggesting macular edema.

#### 2.6.2. Criteria for dose reduction and discontinuation of neoadjuvant chemotherapy

Dose reduction of gemcitabine to 800 mg/m^2^ and nab-paclitaxel to 100 mg/m^2^ is required if patients exhibit any of the following events: neutrophil count < 500/mm^3^; platelet count < 50,000/mm^3^; or Grade 3 febrile neutropenia, diarrhea, anorexia, nausea, vomiting, mucositis, rash, or peripheral sensory/motor neuropathy. Dose reduction of gemcitabine to 600 mg/m^2^ and nab-paclitaxel to 75 mg/m^2^ will be required if patients experience the same event twice. If further dose reduction is required, protocol treatment will be discontinued. If protocol treatment is halted for more than 21 days because of treatment-associated adverse events, protocol treatment will be discontinued.

#### 2.6.3. Surgery

After two cycles of neoadjuvant chemotherapy, enhanced CT will be performed. Surgical resection will be performed if the imaging evaluation suggests R0 resection is possible. An additional one or two cycles of GnP before surgery will be allowed depending on the physicians’ or surgeons’ judgments.

#### 2.6.4. Adjuvant chemotherapy

Adjuvant chemotherapy with S-1 or gemcitabine will be recommended, but not required, in the protocol treatment.

### 2.7. Data collection

Data will be recorded for each patient on a case report form by the investigators of participating institutions and sent to the data center (CReS Kyushu). The schedules of data collection are summarized in ***Table 1***. Patient quality of life will be assessed using EQ-5D-5L, a patient-reported outcomes instrument that can evaluate quality of life, regardless of disease, via six questions.

**Table 1:** Study schedule.


Required measurements	Before registration	During protocol treatment											Discontinuation of protocol treatment

		Chemotherapy								Surgery			
	
		Course	1			2			If chemotherapy is continued	Before surgery	During surgery	28 days after surgery	
	
		Day	1	8	15	29	36	43					

Patient characteristics	○												

Performance status	○		○	○	○	○	○	○	○	○			

Symptoms	○		○	○	○	○	○	○	○	○			

Blood count	○		○	○	○	○	○	○	○	○			

Blood biochemistry	○		○	○	○	○	○	○	○	○			

CEA, CA19-9	○								○	○			

ECG	○												

QOL assessment	○										○	○	○

Contrast CT scan	○									○	○		

Administration record													

Gemcitabine			○	○	○	○	○	○	○				

Nab-paclitaxel			○	○	○	○	○	○	○				

Adverse events				○	○	○	○	○	○	○			

Operative findings											○		

Postoperative complications												○	

Pathological findings												○	


CEA, carcinoembryonic antigen; CA19-9, carbohydrate antigen 19-9; ECG, electrocardiogram; QOL, quality of life; CT, computed tomography.

### 2.8. Central review

To confirm that patients have BRPC-A, central review of MDCT images will be performed.

### 2.9. Statistical analysis

This study is designed as a prospective single-arm phase II trial to evaluate the safety and efficacy of the GnP regimen as neoadjuvant chemotherapy for BRPC-A. The primary endpoint is the R0 resection rate. The R0 resection rate after neoadjuvant therapy for BRPC-A has not been fully elucidated. According to a meta-analysis of neoadjuvant treatment for BRPC, the R0 resection rate after neoadjuvant therapy for BRPC, regardless of arterial involvement, was 57.4% [12]. Therefore, we assume that the R0 resection rate after neoadjuvant treatment for BRPC with arterial involvement will be 30%–50%. The estimated sample size is 35, based on an expected R0 resection rate of 50%, threshold of 30%, alpha error of 0.05, and beta error of 0.2. Because it is anticipated that 5% of registered patients will be ineligible for participation, the planned sample size for this study is 37.

## 3. Ethics and Dissemination

This study protocol was approved by the institutional review board of Kyushu University (no 181.). Written informed consent will be obtained from all participants. The results will be published in a peer-reviewed journal and will be presented at medical meetings.
